# Estimating the burden of iron deficiency among African children

**DOI:** 10.1186/s12916-020-1502-7

**Published:** 2020-02-27

**Authors:** John Muthii Muriuki, Alexander J. Mentzer, Emily L. Webb, Alireza Morovat, Wandia Kimita, Francis M. Ndungu, Alex W. Macharia, Rosie J. Crane, James A. Berkley, Swaib A. Lule, Clare Cutland, Sodiomon B. Sirima, Amidou Diarra, Alfred B. Tiono, Philip Bejon, Shabir A. Madhi, Adrian V. S. Hill, Andrew M. Prentice, Parminder S. Suchdev, Alison M. Elliott, Thomas N. Williams, Sarah H. Atkinson

**Affiliations:** 1 Centre for Geographic Medicine Research-Coast, KEMRI-Wellcome Trust Research Programme, Kenya Medical Research Institute (KEMRI), Kilifi, Kenya; 2 KEMRI – Wellcome Trust Research Programme – Accredited Research Centre, Open University, Kilifi, Kenya; 3 Wellcome Centre for Human Genetics, Nuffield Department of Medicine, University of Oxford, Oxford, UK; 4 Li Ka Shing Centre for Health Information and Discovery, Big Data Institute, University of Oxford, Oxford, UK; 5 MRC Tropical Epidemiology Group, Department of Infectious Disease Epidemiology, London School of Hygiene and Tropical Medicine, London, UK; 6 Department of Clinical Biochemistry, Oxford University Hospitals, Oxford, UK; 7 Centre for Tropical Medicine and Global Health, Nuffield Department of Medicine, University of Oxford, Oxford, UK; 8 Medical Research Council/Uganda Virus Research Institute and London School of Hygiene and Tropical Medicine Uganda Research Unit, Entebbe, Uganda; 9 Medical Research Council: Respiratory and Meningeal Pathogens Research Unit, Faculty of Health Sciences, University of the Witwatersrand, Johannesburg, South Africa; 10 Groupe de Recherche Action en Sante (GRAS), Ouagadougou 06, 06 BP 10248 Burkina Faso; 11 Centre for Clinical Vaccinology and Tropical Medicine and the Jenner Institute Laboratories, University of Oxford, Oxford, UK; 12 Medical Research Council Unit The Gambia at London School of Hygiene and Tropical Medicine, Banjul, The Gambia; 13 Department of Pediatrics, Emory University and Emory Global Health Institute, Atlanta, GA USA; 14 Department of Clinical Research, London School of Hygiene and Tropical Medicine, London, UK; 15 Department of Medicine, Imperial College, London, UK; 16 Department of Paediatrics, University of Oxford, Oxford, UK

**Keywords:** Iron deficiency, Ferritin, Transferrin saturation, Inflammation, Malaria, African children

## Abstract

**Background:**

Iron deficiency (ID) is a major public health burden in African children and accurate prevalence estimates are important for effective nutritional interventions. However, ID may be incorrectly estimated in Africa because most measures of iron status are altered by inflammation and infections such as malaria. Through the current study, we have assessed different approaches to the prediction of iron status and estimated the burden of ID in African children.

**Methods:**

We assayed iron and inflammatory biomarkers in 4853 children aged 0–8 years from Kenya, Uganda, Burkina Faso, South Africa, and The Gambia. We described iron status and its relationship with age, sex, inflammation, and malaria parasitemia. We defined ID using the WHO guideline (ferritin < 12 μg/L or < 30 μg/L in the presence of inflammation in children < 5 years old or < 15 μg/L in children ≥ 5 years old). We compared this with a recently proposed gold standard, which uses regression-correction for ferritin levels based on the relationship between ferritin levels, inflammatory markers, and malaria. We further investigated the utility of other iron biomarkers in predicting ID using the inflammation and malaria regression-corrected estimate as a gold standard.

**Results:**

The prevalence of ID was highest at 1 year of age and in male infants. Inflammation and malaria parasitemia were associated with all iron biomarkers, although transferrin saturation was least affected. Overall prevalence of WHO-defined ID was 34% compared to 52% using the inflammation and malaria regression-corrected estimate. This unidentified burden of ID increased with age and was highest in countries with high prevalence of inflammation and malaria, where up to a quarter of iron-deficient children were misclassified as iron replete. Transferrin saturation < 11% most closely predicted the prevalence of ID according to the regression-correction gold standard.

**Conclusions:**

The prevalence of ID is underestimated in African children when defined using the WHO guidelines, especially in malaria-endemic populations, and the use of transferrin saturation may provide a more accurate approach. Further research is needed to identify the most accurate measures for determining the prevalence of ID in sub-Saharan Africa.

**Electronic supplementary material:**

The online version of this article (10.1186/s12916-020-1502-7) contains supplementary material, which is available to authorized users.

## Background

Iron deficiency (ID) is the commonest nutrient deficiency affecting over 2 billion people worldwide and is a major public health burden in African children [1, 2]. ID has been associated with impaired brain development and long-term impairment of behavioral and cognitive performance [3]. In sub-Saharan Africa, ID anemia (IDA) is the leading cause of years lived with disability (YLD) in 30 of the 46 countries [2]. However, despite its detrimental effects on health, the true burden of ID in African children remains largely unknown because of the complex interactions of the biochemical markers of ID with inflammation and infections including malaria [4, 5].

Reliable and accurate estimation of the prevalence of ID is essential in planning, monitoring, and targeting effective interventions. The gold standard method for estimating iron status is to stain bone marrow aspirate for iron, but this is invasive and impractical in population surveys [6]. To define ID in areas with high burden of infectious diseases, the World Health Organization (WHO) recommends the use of low ferritin concentrations (< 12 μg/L in children < 5 years or < 15 μg/L in children ≥ 5 years), with an arbitrarily higher cut-off of ferritin (< 30 μg/L) in children < 5 years with inflammation (defined as C-reactive protein (CRP) > 5 mg/L) [7, 8]. Ferritin reflects body iron stores and has standardized laboratory assays and established cut-offs; however, its synthesis is also highly upregulated by inflammatory cytokines [9] and by malaria, even in the absence of inflammation [10]. We therefore hypothesized that the WHO definition of ID may underestimate the prevalence of ID in areas with high burdens of inflammation and malaria.

A range of alternative markers of iron status have been proposed to determine iron status, but these also have limitations. Soluble transferrin receptor (sTfR) is only mildly increased during the inflammatory response [11], but its utility is complicated in African populations since it is upregulated by malaria, even in asymptomatic infection, and by hemolytic conditions such as sickle cell disease, thalassemia, and glucose-6-phosphate dehydrogenase (G6PD) deficiency. Moreover there are no standardized sTfR reference assays [12–14]. Other iron biomarkers may also be confounded by the effects of inflammation or malaria [8]. Although hemoglobin defines anemia, its usefulness in defining ID is limited due to its low specificity since the causes of anemia are multifactorial and hemoglobin levels only decline in late-stage deficiency [15]. Estimating iron status in African children is therefore challenging, although a number of approaches have been proposed to account for the effects of inflammation and malaria, including using higher ferritin cut-offs, or excluding individuals with raised inflammatory markers [4]. A regression-correction approach, which accounts for the linear effects of inflammatory markers and/or malaria on iron biomarkers, as proposed by the Biomarkers Reflecting Inflammation and Nutritional Determinants of Anemia (BRINDA) project, appears to reflect iron status more accurately [10, 16].

In the current study, we have measured a wide range of iron markers in 4853 African children and described their relationship with age, sex, underweight, inflammation, and malaria parasitemia. We then utilized the regression-correction approach proposed by BRINDA [10, 16] to predict what the ferritin level would have been in the absence of inflammation and malaria, and then used these predicted values to estimate the prevalence of ID in African children. We then compared the regression-corrected prevalence of ID to the prevalence of WHO-defined ID. Finally, we assessed the diagnostic utility of various iron markers in predicting ID using an approach based on the BRINDA regression-correction method as a gold standard.

## Methods

### Study population

This study included community-based cohorts from Kenya, Uganda, Burkina Faso, South Africa, and The Gambia.

#### Kilifi, Kenya

Participants were members of an ongoing rolling cohort evaluating malaria immunity in children as described elsewhere [17]. Within this cohort, children were followed up to 8 years with weekly follow-ups and annual cross-sectional surveys during which anthropometry measurements and blood samples were taken. Iron and inflammatory biomarkers as well as malaria parasitemia were measured from blood samples collected at a single cross-sectional survey based on the availability of plasma samples archived at − 80 °C.

#### Entebbe, Uganda

The Entebbe Mother and Baby Study is a prospective birth cohort study that was originally designed as a randomized, double-blind, placebo-controlled trial (ISRCTN32849447) to determine whether anthelminthic treatment during pregnancy and early childhood was associated with differential response to vaccination or incidence of infections such as pneumonia, diarrhea, or malaria [18]. Blood samples were collected at birth and at subsequent annual visits up to age 5 years. Anthropometry and iron/inflammatory biomarkers were measured from a single annual visit based on the availability of stored samples.

#### Banfora, Burkina Faso

The VAC050 ME-TRAP malaria vaccine trial tested the safety, immunogenicity, and efficacy of a viral-vectored prime-boost liver-stage malaria vaccine in infants between the ages of six and 17 months living in the Banfora region of Burkina Faso [19]. Anthropometry and plasma samples were available from the infants at multiple time-points following receipt of the experimental vaccine. Iron and inflammatory biomarkers were assayed from samples collected at time-points close to 12 months of age based on another study looking at the genetics of responses against vaccines delivered as part of the Expanded Programme on Immunization (EPI).

#### Soweto, South Africa

Infants born in Chris Hani Baragwanath Hospital living in Soweto, a non-malaria-endemic region in Johannesburg, South Africa, were recruited from vaccine trials coordinated by the Respiratory and Meningeal Pathogens Unit (http://www.rmpru.com/) [20]. This study used plasma samples collected at 12 months of age in infants who had received all of their EPI vaccines up to 6 months of age. Anthropometry and hemoglobin concentrations were not measured in this cohort.

#### West Kiang, The Gambia

All children aged 2 to 6 years and living in the West Kiang region of The Gambia were recruited during the malaria season to assess the effects of genetic variants on hemoglobin concentrations and iron status [21]. Iron biomarkers, anthropometric measurements, and malaria parasitemia data were measured at a cross-sectional survey at the start of a malaria season.

### Laboratory procedures

The assayed biomarkers of iron (plasma ferritin, sTfR, hepcidin, serum iron, transferrin, unsaturated iron-binding capacity (UIBC), zinc protoporphyrin (ZPP), and hemoglobin) and inflammation (CRP and α_1_-antichymotrypsin (ACT)) are shown in Additional file 1: Table S1. UIBC, ACT, and ZPP were only measured in Gambian children. The Gambian hepcidin values were harmonized by converting to the old DRG hepcidin assay values and then to the new highly sensitive DRG hepcidin assay values [22]. In Uganda, hemoglobin concentrations were adjusted for an altitude of > 1000 m above sea level (by subtracting 0.2 g/dL) [23]. *Plasmodium falciparum* and other *Plasmodium* species were examined using Giemsa-stained thick and thin blood smears. All assays are based on single measurements per child.

### Definitions

Inflammation was defined as CRP > 5 mg/L or ACT > 0.6 g/L [8]. ID was defined using the WHO recommended definition as either (i) plasma ferritin < 12 μg/L in the absence of inflammation or < 30 μg/L in the presence of inflammation in children < 5 years or (ii) plasma ferritin < 15 μg/L in children ≥ 5 years [7]. Body iron stores (BIS) were calculated as proposed by Cook et al. as − [log10((sTfR in mg/L × 1000)/ferritin in μg/L) − 2.8229]/0.1207 [24]. Ferritin index, a marker of bone marrow iron depletion, was calculated as sTfR in mg/L/log10(ferritin in μg/L) [25]. Transferrin saturation (TSAT) was calculated as (serum iron in μmol/L/transferrin in g/L) × 25.1) × 100 in Kenya and Burkina Faso or as (serum iron in μmol/L/ UIBC in μmol/L + serum iron in μmol/L) × 100 in The Gambia [26]. Serum iron measurements for calculation of TSAT were not available in Uganda and South Africa because plasma samples were stored in ethylenediaminetetraacetic acid (EDTA), which chelates iron. Anemia was defined as Hb < 11 g/dL in children aged < 5 years, or hemoglobin < 11.5 g/dL in children ≥ 5 years, while IDA was defined as the presence of both ID and anemia [27]. Malaria parasitemia was defined as microscopy confirmed *P. falciparum* parasitemia at any density. Underweight was defined as weight-for-age *z*-score < − 2 using the WHO Growth Reference Standards [28].

### Statistical analyses

All analyses were conducted using STATA 13.0 (StataCorp., College Station, TX). Iron biomarkers (except transferrin, hemoglobin and BIS) were ln-transformed to normalize their distributions. Differences in biomarker means between age groups were tested using two-tailed Student’s *t* tests assuming unequal variance. We fitted univariable and multivariable linear regression models to determine the associations between iron biomarkers and age, sex, underweight, inflammation, and malaria parasitemia. Where analyses were pooled, Gambian sTfR, TSAT, BIS, and ferritin index were excluded since different assays were used in this population meaning that values were not directly comparable with those from other cohorts (Additional file 1: Table S1). All *p* values reflect two-tailed tests and a *p* value of < 0.05 was considered significant.

#### Regression correction

Following analyses of predictors of iron status, we then aimed to estimate the prevalence of ID by adjusting for the effects of inflammation and malaria on ferritin levels using a regression-correction approach as developed by BRINDA [10, 16]. We used these estimates as our gold standard. The regression-correction approach followed a three-step process. In the first step, internal reference values for inflammatory markers (CRP or ACT) were defined as the 10th percentile. To avoid overcorrection for very low levels of inflammatory markers, only participants with CRP or ACT values above the 10th percentile (0.2 mg/L and 0.3 g/L for unlogged CRP and ACT respectively) had their ferritin values subtracted from observed values in Eqs. (1)–(3) below [10]. In the second step, univariable linear regression models were applied to the full dataset, with ferritin as the dependent variable, to estimate regression coefficients for the crude association between inflammatory marker level and ferritin (*β*_1_), and for the crude association between malaria and ferritin (*β*_2_), and multivariable linear regression was applied to estimate adjusted regression coefficients for associations between inflammatory marker level and ferritin (*β*_3_) and between malaria parasitemia and ferritin (*β*_4_). In the third step, the regression coefficients estimated in step 2 were used to calculate adjusted ferritin values using Eq. (1), (2), or (3). For the purposes of comparison, Eq. (1) was applied to adjust for the inflammatory marker only, Eq. (2) for malaria parasitemia only, and Eq. (3) for both inflammatory marker and malaria parasitemia. Ferritin and inflammatory markers were applied in the equations after ln-transformation.

1 $$ {\mathrm{Ferritin}}_{\mathrm{adjusted}1}={\mathrm{Ferritin}}_{\mathrm{unadjusted}}-{\beta}_1\left(\mathrm{CRP}\ \mathrm{or}\ {\mathrm{ACT}}_{\mathrm{obs}}-\mathrm{CRP}\ \mathrm{or}\ {\mathrm{ACT}}_{\mathrm{ref}}\right) $$

2 $$ {\mathrm{Ferritin}}_{\mathrm{adjusted}2}={\mathrm{Ferritin}}_{\mathrm{unadjusted}}-{\beta}_2\mathrm{malaria} $$

3 $$ {\mathrm{Ferritin}}_{\mathrm{adjusted}3}={\mathrm{Ferritin}}_{\mathrm{unadjusted}}-{\beta}_3\left(\mathrm{CRP}\ \mathrm{or}\ {\mathrm{ACT}}_{\mathrm{obs}}-\mathrm{CRP}\ \mathrm{or}\ {\mathrm{ACT}}_{\mathrm{ref}}\right)-{\beta}_4\mathrm{malaria} $$

where “obs” is the observed value and “ref” is the reference value.

We then defined ID using the regression-corrected unlogged ferritin (i.e., adjusted for the effects of inflammation and malaria) using the same thresholds that were applied to the uncorrected ferritin levels in the WHO recommendations (i.e., ferritin < 12 μg/L in children < 5 years or < 15 μg/L in children aged ≥ 5 years [7]) and compared changes in prevalence of ID using McNemar’s chi-square test of consistency. In further models, we additionally corrected ferritin levels for age, sex, and underweight. We also applied regression-correction for inflammation and malaria to other markers of iron status including sTfR, hepcidin, BIS, ferritin index, and ZPP. We then tested the diagnostic utility of the uncorrected biomarkers in predicting ID regression-corrected for inflammation and malaria as the “gold standard.” We used receiver operating characteristics curve (ROC) analyses using regression-corrected ID as a binary classifier for identifying the optimal cut-off values of the continuous iron biomarkers. We defined the optimal cut-off value as a point on the curve where the Youden index (sensitivity + specificity − 1) is maximum [29].

## Results

### Characteristics of study participants

A total of 4853 children, 1484 Kenyan, 1374 Ugandan, 348 Burkinabe, 894 South African, and 753 Gambian, aged between birth and 8 years were included in this study. Table 1 shows the characteristics of study participants in the five African cohorts. The prevalence of malaria parasitemia was highest in Kenya (21.9%) and Burkina Faso (20.6%) and lower in Uganda (6.8%). Similarly, the prevalence of inflammation was high in Burkina Faso (33.9%) and Kenya (27.3%), but lower in South Africa (17.6%) and The Gambia (14.9%).

**Table 1:** Characteristics of study participants by cohort

Characteristic	Kenya *n* = 1484	Uganda *n* = 1374	Burkina Faso *n* = 348	South Africa *n* = 894	The Gambia *n* = 753	Pooled *n* = 4853
Age in years, *n*/total (%)						
< 1	321/1484 (21.6)	26/1374 (1.9)	21/348 (6.0)	475/894 (53.1)	0/753 (0.0)	843/4853 (17.4)
1–< 2	597/1484 (40.2)	459/1374 (33.4)	185/348 (53.2)	418/894 (46.8)	16/753 (2.1)	1675/4853 (34.5)
2–< 3	170/1484 (11.5)	622/1374 (45.3)	142/348 (40.8)	1/894 (0.1)	188/753 (25.0)	1123/4853 (23.1)
3–< 4	159/1484 (10.7)	176/1374 (12.8)	0	0	201/753 (26.7)	536/4853 (11.0)
4–8	237/1484 (16.0)	91/1374 (6.6)	0	0	348/753 (46.2)	676/4853 (13.9)
Age in years, median (IQR)	1.7 (1.1, 3.1)	2.0 (2.0, 3.0)	1.9 (1.6, 2.2)	1.0 (1.0, 1.0)	3.8 (2.9, 4.9)	2.0 (1.0, 3.0)
Gender: females, *n*/total (%)	726/1484 (48.9)	678/1374 (49.3)	173/348 (49.7)	449/894 (50.2)	331/753 (46.8)	2378/4853 (49.0)
Underweight^*^, *n*/total (%)	114/429 (26.6)	113/1368 (8.3)	60/327 (18.4)	n/a	150/593 (25.3)	437/2717 (16.1)
Inflammation^†^, *n*/total (%)	392/1437 (27.3)	316/1337 (23.6)	112/330 (33.9)	157/894 (17.6)	112/753 (14.9)	1089/4751 (22.9)
Malaria^‡^, *n*/total (%)	262/1199 (21.9)	92/1353 (6.8)	66/321 (20.6)	n/a	84/751 (11.2)	504/4518 (11.2)
Parasite density, median (IQR)	2295 (604, 6619)	122 (36, 350)	3714 (887, 18,103)	n/a	n/a	1386 (224, 5523)

*n/a* not available, *IQR* interquartile range

*Underweight was defined as weight-for-age *z*-score < − 2. Not measured in the South African cohort

†Inflammation was defined as C-reactive protein > 5 mg/L or α1-antichymotrypsin > 0.6 g/dL (in The Gambia)

‡Malaria was defined as *P. falciparum* parasitemia and parasite density as parasites/μL. Children in South Africa were not exposed to malaria and parasite density data was not available for The Gambia. Pooled parasite density did not include South African or Gambian data

### Distribution of iron status and anemia

Table 2 shows the prevalence of ID and anemia, and concentrations of the individual iron biomarkers by study cohort. Based on the WHO recommended definition, the prevalence of ID was highest in South African children (41.9%) and lowest in The Gambia (21.7%) and affected about a third of children in each of Kenya (35.4%), Uganda (34.6%), and Burkina Faso (35.5%). Anemia was present in 87.0% of children in Burkina Faso, 70.0% in Kenya, 60.1% in The Gambia, and 49.7% in Uganda.

**Table 2:** Distribution of iron status and anemia by study cohort

	Kenya			Uganda			Burkina Faso			South Africa			The Gambia			Pooled^*^		
Category	*n*/total	%	95% CI	*n*/total	%	95% CI	*n*/total	%	95% CI	*n*/total	%	95% CI	*n*/total	%	95% CI	*n*/total	%	95% CI
Iron deficiency^†^	499/1408	35.4	32.9, 37.9	438/1267	34.6	31.9, 37.2	115/324	35.5	30.3, 40.7	375/894	41.9	38.7, 45.2	163/752	21.7	18.7, 24.6	1590/4645	34.2	32.9, 35.6
Anemia^‡^	609/870	70.0	66.9, 73.1	652/1312	49.7	47.0, 52.4	288/331	87.0	83.4, 90.6	n/a	n/a	n/a	448/746	60.1	56.5, 63.6	1997/3259	61.3	59.6, 62.9
Iron deficiency anemia^§^	207/833	24.8	21.9, 27.8	255/1209	21.1	18.8, 19.8	96/309	31.1	25.9, 36.3	n/a	n/a	n/a	123/745	16.5	13.8, 19.2	681/3096	22.0	20.5, 23.5
Biomarker	*n*	Mean	95% CI	*n*	Mean	95% CI	*n*	Mean	95% CI	*n*	Mean	95% CI	*n*	Mean	95% CI	*n*	Mean	95% CI
Hemoglobin, g/dL	870	10.2	10.1, 10.3	1312	9.1	9.0, 9.2	331	9.6	9.4, 9.7	n/a	n/a	n/a	746	10.7	10.6, 10.8	3259	10.5	10.5, 10.6
Ferritin, μg/L	1408	21.8	20.6, 23.1	1267	20.8	19.6, 22.0	324	22.2	19.7, 24.9	894	14.9	14.0, 15.9	752	25.1	23.6, 26.8	4645	20.5	19.9, 21.1
sTfR, mg/L	1467	17.9	17.5, 18.3	1343	6.7	6.5, 7.0	342	17.7	16.7, 18.7	893	11.2	10.9, 11.5	661	3.4	3.4, 3.5	4045	11.7	11.4, 11.9
Hepcidin, μg/L	1373	5.8	5.4, 6.2	1333	6.8	6.4, 7.2	309	5.3	4.6, 6.3	878	7.7	7.1, 8.4	709	6.0	5.3, 6.7	4602	6.4	6.2, 6.6
BIS, mg/kg^**^	1393	−0.8	−1.0, −0.5	1241	2.5	2.2, 2.8	322	−0.6	−1.1, −0.1	893	−0.4	−0.7, −0.2	660	5.7	5.4, 6.0	3849	0.4	0.2, 0.5
Ferritin index^††^	1389	14.4	13.9, 14.9	1234	5.5	5.3, 5.8	322	13.9	12.8, 15.1	881	10.1	9.7, 10.6	660	2.6	2.5, 2.7	3826	9.7	9.5, 10.0
Serum iron, μmol/L	1428	6.5	6.3, 6.7	n/a	n/a	n/a	337	6.0	5.7, 6.4	n/a	n/a	n/a	737	8.6	8.3, 8.9	1765	6.4	6.2, 6.6
Transferrin, g/L	1409	2.8	2.7, 2.8	1333	2.8	2.6, 2.7	327	2.7	2.6, 2.8	894	2.7	2.7, 2.8	n/a	n/a	n/a	3963	2.8	2.7, 2.8
TSAT, %^‡‡^	1386	9.4	9.0, 9.8	n/a	n/a	n/a	325	8.9	8.3, 9.7	n/a	n/a	n/a	734	12.8	12.3, 13.3	1711	9.3	9.0, 9.6
ZPP, μmol/mol heme	n/a	n/a	n/a	n/a	n/a	n/a	n/a	n/a	n/a	n/a	n/a	n/a	751	85.9	82.4, 89.5	751	85.9	82.4, 89.5
MCV, fL	499	65.7	65.0, 66.5	1305	71.0	70.6, 71.5	n/a	n/a	n/a	n/a	n/a	n/a	741	75.6	75.1, 76.1	2545	71.2	70.9, 71.6

*n/a* not available, *BIS* body iron store, *sTfR* soluble transferrin receptors, *TSAT* transferrin saturation, *ZPP* zinc protoporphyrin, *MCV* mean corpuscular volume

The mean values are geometric means except for BIS, transferrin, hemoglobin, and MCV which are arithmetic means

*Pooled values for sTfR, BIS, ferritin index, iron, transferrin, and TSAT, exclude The Gambia since markers were measured using different assays compared to the other cohorts

†Iron deficiency defined using the WHO definition as ferritin < 12 μg/L or < 30 μg/L in presence of inflammation (C-reactive protein> 5 mg/L or α1-antichymotrypsin > 0.6 g/dL) in children < 5 years or < 15 μg/L in children ≥ 5 years [8]

‡Anemia was defined as hemoglobin < 11 g/dL in children aged 0 to 5 years or hemoglobin < 11.5 g/dL in children above 5 years [27]. Hemoglobin measurements unavailable in South Africa

§Iron deficiency anemia defined as iron deficiency and anemia [27]

**Body iron store was calculated using the ratio of soluble transferrin receptors and ferritin concentrations, −(log10((sTfR × 1000)/ferritin) − 2.8229)/0.1207 [24]

††Ferritin index was defined as sTfR/log10 ferritin [25]

‡‡TSAT was calculated as ((iron (μmol/L)/transferrin (g/L) × 25.1) × 100) except in The Gambia where it was calculated using iron and unsaturated iron binding capacity [26]. Iron and TSAT missing in Uganda and South Africa and transferrin missing in The Gambia

### Age, sex, and nutritional differences in iron status

**Fig. 1:**
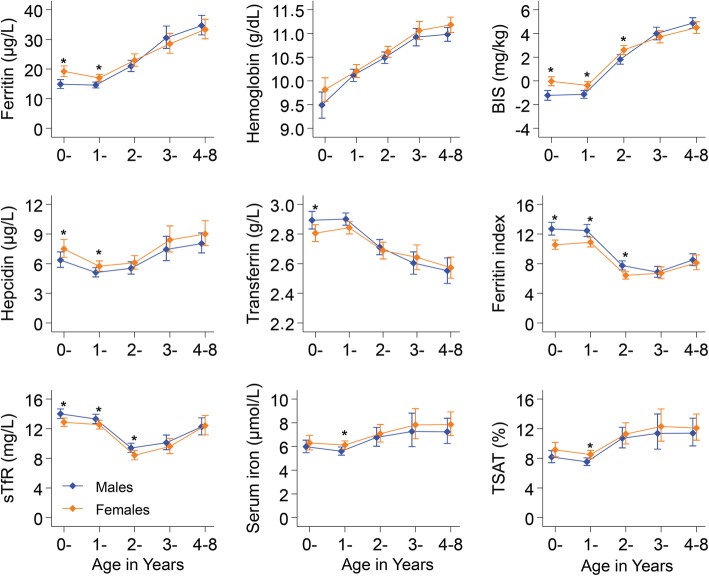
Geometric means for different iron biomarkers by age in years and sex. Orange indicates females and blue males. Error bars indicate 95% confidence intervals. Star indicates Student’s *t* test *p* value < 0.05 for mean differences between sex. BIS, body iron stores; sTfR, soluble transferrin receptor; TSAT, transferrin saturation

Concentrations of ferritin, hepcidin, BIS, and TSAT decreased during the first year of life and increased thereafter, indicating that ID is most prevalent at about 1 year of age (Fig. 1). Male infants were more iron deficient than female infants for each of the different measures of iron status, although hemoglobin concentrations did not differ by sex. Sex-specific differences were not observed beyond 3 years of age. The odds of malaria parasitemia and underweight increased with age but did not differ by sex, while underweight children were more likely to have inflammation and malaria parasitemia (Additional file 2: Table S2). Underweight was associated with reduced hemoglobin levels, BIS, and higher sTfR levels and ferritin index in models adjusted for age, sex, study site, inflammation, and malaria parasitemia (Additional file 4: Figure S1).

### Associations between inflammation and malaria and iron status

**Fig. 2:**
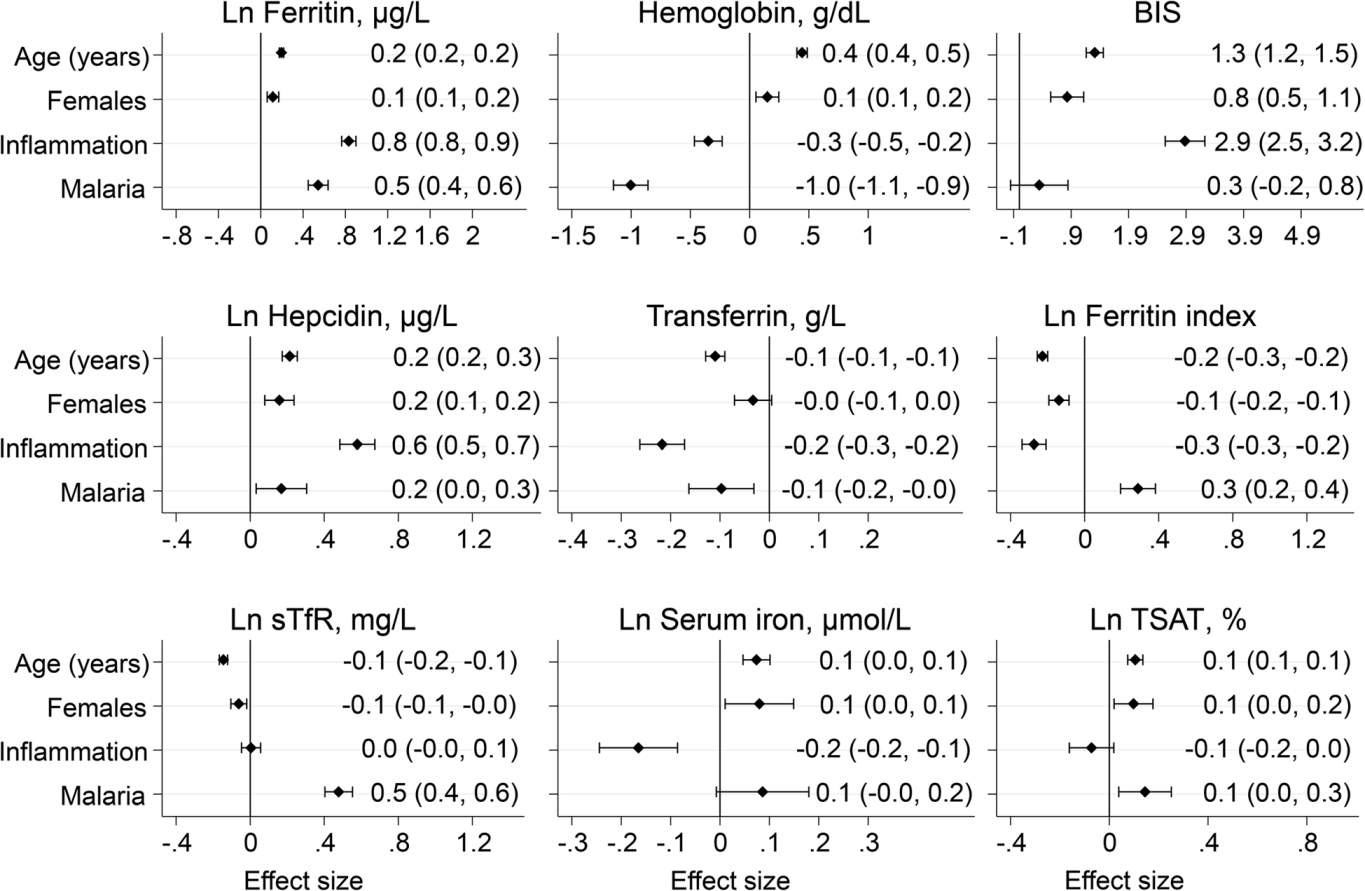
Predictors of individual iron biomarkers. Effect size represents coefficient from multivariable linear regression model with the iron biomarker as the outcome variable. Models were adjusted for age, sex, study site, inflammation, and malaria. Iron biomarkers were ln-transformed except hemoglobin, transferrin, and BIS. Error bars indicate 95% confidence intervals and values indicate effect size (95% CI). Inflammation was defined as C-reactive protein > 5 mg/L or α1-antichymotrypsin > 0.6 g/dL (in The Gambia). Malaria was defined as *P. falciparum* parasitemia. BIS, body iron stores; sTfR, soluble transferrin receptor; TSAT, transferrin saturation

We then tested for associations between inflammation/malaria parasitemia and individual markers of iron status in multivariable models adjusted for age, sex, study site, inflammation and malaria parasitemia (Fig. 2). Notably, both inflammation and malaria parasitemia were independently associated with substantially increased ferritin levels. Inflammation was also independently associated with increased BIS, hepcidin, and ZPP levels and decreased hemoglobin, transferrin, ferritin index, and serum iron levels (Fig. 2 and Additional file 4: Figure S1). Malaria parasitemia was also independently associated with increased sTfR, hepcidin and ferritin index, and decreased hemoglobin and transferrin after adjustment for inflammation, age, sex, and study site. Overall, TSAT was least affected by both inflammation and malaria, particularly after additional adjustment for underweight (Additional file 4: Figure S1).

### Estimating the regression-corrected prevalence of iron deficiency

**Fig. 3:**
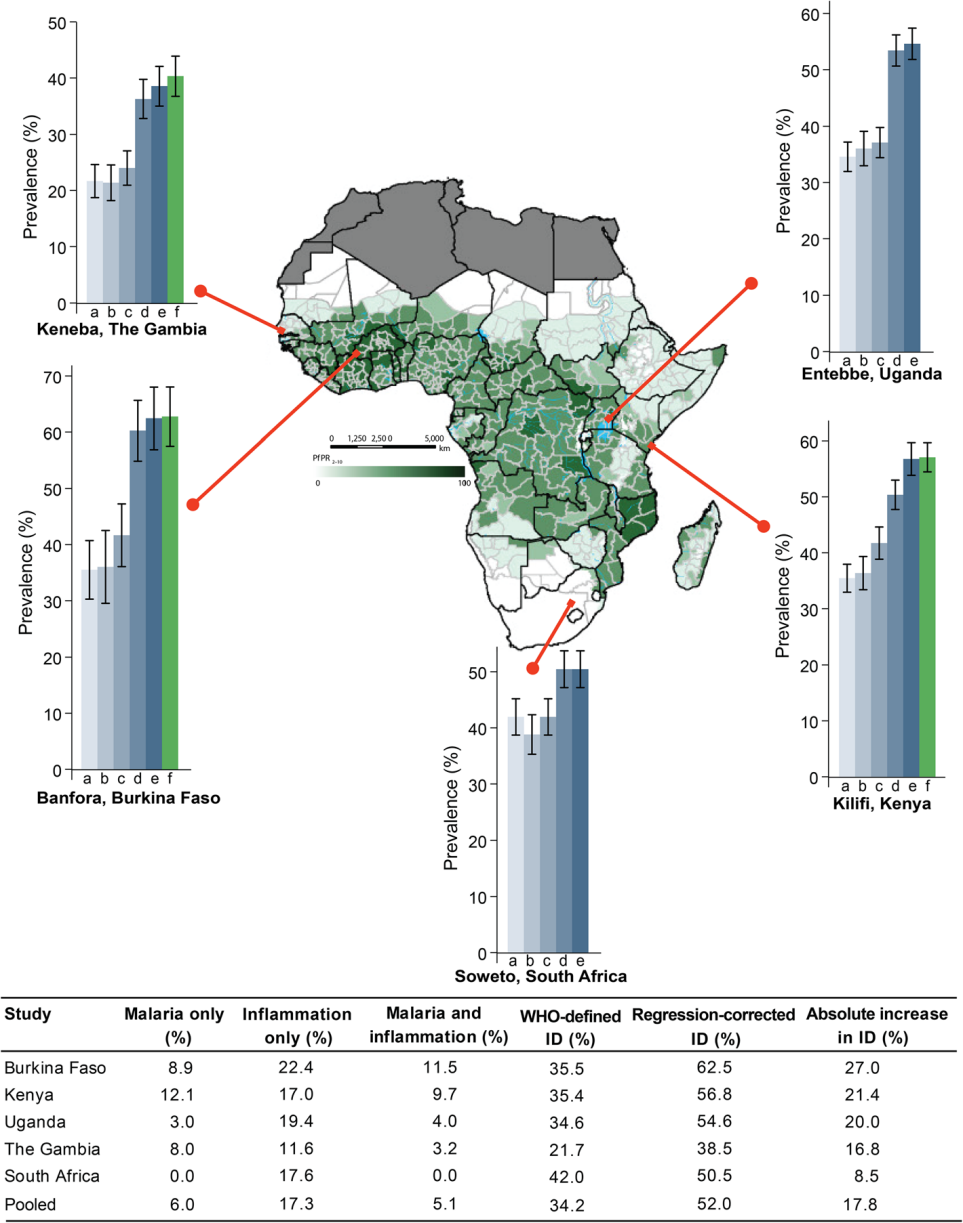
Prevalence of estimated iron deficiency across the study sites. The map shows the predicted posterior predictions of age-standardized *P. falciparum* prevalence (PfPR_2–10_) as previously published by Snow et al. [30]. Map was reproduced with permission. Graph letter “a” indicates prevalence of iron deficiency using the WHO definition, “b” excluding children with inflammation, “c” adjusting for malaria only, “d” adjusting for inflammation only, “e” adjusting for both malaria and inflammation, and “f” using transferrin saturation cut-off of < 11%. Values indicate prevalence. Malaria only indicates percentage of children with malaria parasitemia without inflammation, inflammation only as percentage with inflammation and no parasitemia, and malaria and inflammation as percentage with both parasitemia and inflammation. Absolute increase in iron deficiency was calculated as the difference between regression-corrected prevalence (corrected for both malaria and inflammation) and WHO-defined prevalence. Error bars indicate 95% confidence intervals

Ferritin levels were then adjusted for inflammation and malaria using the regression-correction approach proposed by BRINDA. Figure 3 shows the prevalence of ID mapped on the African malaria map for the period 2010–2015 [30]. Excluding children with inflammation resulted in a similar prevalence of ID as WHO-defined ID. Adjustment of ferritin levels for inflammation alone substantially increased the prevalence of ID compared to adjustment for malaria alone, while adjustment for both malaria and inflammation led to a small further increase especially in Kenyan children who had the highest prevalence of malaria without inflammation (Fig. 3). Further adjustments for age, sex, and underweight did not change the prevalence of ID (Additional file 5: Figure S2). The pooled prevalence of ID after adjusting for both inflammation and malaria was 52.0% and the absolute increase in the prevalence of ID for each study site was as follows: Burkina Faso, 27.0%; Kenya, 21.4%; Uganda, 20.0%; The Gambia, 16.8%; and South Africa, 8.5% (Fig. 3). The gap between WHO-defined ID and regression correction was highest in cohorts that had the highest prevalence of malaria and inflammation (Kenya and Burkina Faso) and lowest in malaria-free South Africa. The prevalence of ID defined by other iron biomarkers and by IDA similarly increased following regression-correction for inflammation and malaria (Additional file 3: Table S3).

### Misclassification of iron-deficient children increases with age, inflammation and malaria

**Fig. 4:**
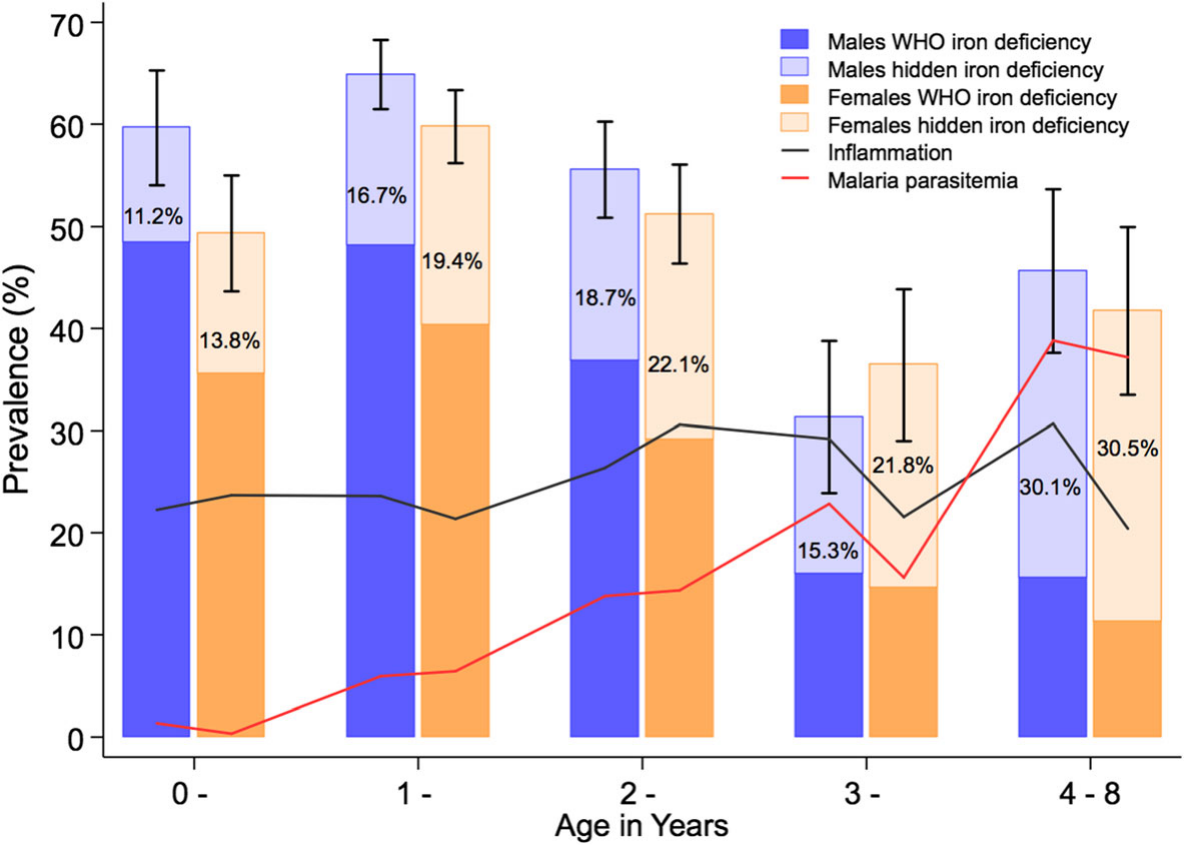
The burden of iron deficiency varies by age, sex, inflammation, and malaria parasitemia. Error bars indicate 95% confidence intervals for prevalence of iron deficiency regression-corrected for inflammation and malaria. Darker colors indicate the WHO definition of iron deficiency while lighter colors show the gap in the prevalence of iron deficiency between the two definitions (referred to as “hidden iron deficiency”). The values in the bars indicate the percentage of children with iron deficiency unaccounted for by the WHO definition. Line plots indicate how the prevalence of inflammation (black) and malaria (red) changed with age

**Fig. 5:**
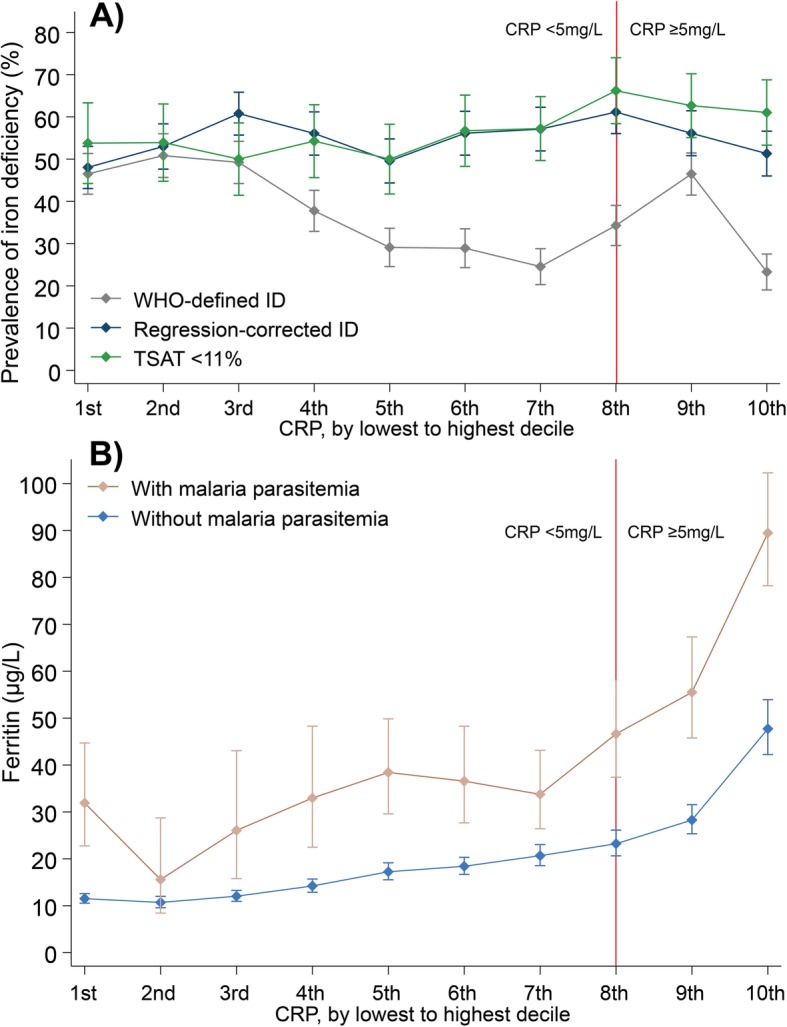
Relationship between the estimated prevalence of iron deficiency and inflammation. **a** How the prevalence of estimates of iron deficiency including WHO-defined ID, regression-corrected ID (corrected for inflammation and malaria), and TSAT < 11%, varied by deciles of C-reactive protein (CRP) and **b** ferritin levels were higher in children with malaria parasitemia compared to those without parasitemia at every CRP decile. Error bars indicate 95% confidence intervals. TSAT, transferrin saturation

The gap between the prevalence of WHO-defined ID and regression-corrected ID increased with age, increasing prevalence of malaria parasitemia (Fig. 4) and with increasing CRP levels (Fig. 5a). Prevalence of regression-corrected ID remained relatively constant over the spectrum of CRP levels, while prevalence of WHO-defined ID decreased linearly above the third decile of CRP (0.4 mg/L), before adjustment for inflammation (CRP > 5 mg/L) (Fig. 5a). Malaria may also contribute to underestimation of prevalence of ID. Malaria-endemic countries had a higher percentage of children misclassified as iron replete (27.0% in Burkina Faso compared to 8.5% in South Africa; Fig. 3). The gap between WHO-defined and regression-corrected prevalence of ID was larger in children with malaria parasitemia compared to those without, regardless of the presence of inflammation (Additional file 6: Figure S3). Children with malaria also had higher ferritin concentrations at every decile of CRP, compared to those without malaria (Fig. 5b).

### Diagnostic utility of iron biomarkers in predicting regression-corrected iron deficiency

**Fig. 6:**
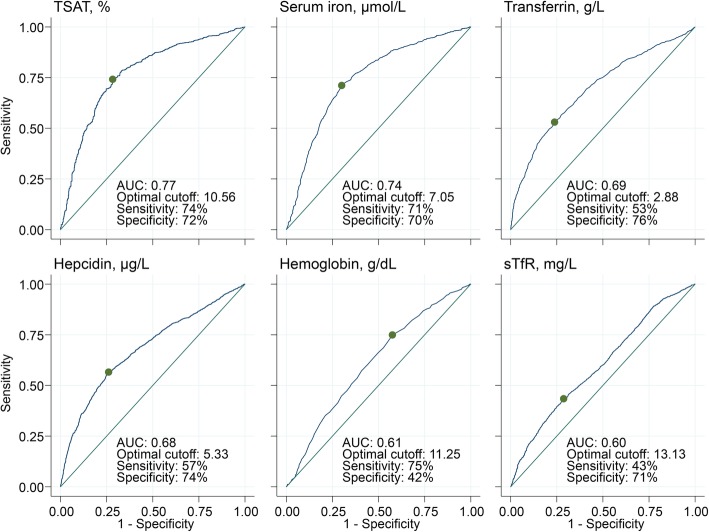
Receiver operating characteristic curves of the utility of iron markers in predicting regression-corrected iron deficiency. The “gold standard” was defined using the WHO definition adjusted for malaria and inflammation using regression correction. Green points indicate Youden’s optimal cut-offs for each marker. Sensitivity and specificity are for the optimal cut-off. TSAT, transferrin saturation; sTfR, soluble transferrin receptor; AUC, area under curve

Finally, we used the regression-corrected ID (corrected for the effects of inflammation and malaria on ferritin levels) as a gold standard to test the diagnostic utility of various markers of iron status (Fig. 6). TSAT outperformed other markers of iron status. For TSAT, we observed an area under curve (AUC) of 0.77 and an optimal cut-off of 10.6, similar to the overall cut-off (11.1) that was obtained from a meta-analysis of cohort-specific optimal cut-offs (Fig. 6 and Additional file 7: Figure S4). We then applied a rounded cut-off of TSAT < 11% and obtained similar prevalence of ID as that obtained using the regression-corrected definition of ID (Fig. 3). TSAT < 11% also performed well across the spectrum of CRP levels with only a modest increase in prevalence of ID compared to regression-corrected ID during inflammation (Fig. 5a). Other iron biomarkers did not perform as well in predicting regression-corrected ID. Hemoglobin concentrations had an AUC of 0.61 and an optimal cut-off of 11.25 g/dL with a sensitivity of 75%, but low specificity of 42%, whilst sTfR concentrations had low sensitivity (43%) in predicting ID corrected for inflammation and malaria (Fig. 6).

## Discussion

Through this study, we have described iron status and estimated the prevalence of ID in more than 4800 children living across the African continent using a wide range of iron biomarkers. Among infants, ID increased from birth to approximately 1 year, and then decreased with increasing age. Males were generally more iron deficient than females up to 3 years of age. Underweight children had increased ID and erythropoietic drive. Inflammation and malaria parasitemia were associated with independent and substantial increases in ferritin concentrations and were also associated with other biomarkers of iron status. We found that WHO-defined ID underestimated the burden of ID in African children compared to regression correction, which predicts ferritin levels in the absence of inflammation and malaria. Of the other iron markers that we tested, TSAT had the best diagnostic properties when compared against the gold standard of regression correction.

Age, sex, and being underweight were associated with iron status in young African children. We found that iron stores decreased rapidly during infancy and reached a nadir at around 1 year of age, supporting the argument that newborns have higher iron stores that are prenatally accumulated but decrease with demand for iron during rapid growth and low iron supply from breast milk and complementary foods [31]. Iron status improved during childhood, perhaps due to diversifying diets and reducing growth rates post-infancy. Female infants were less iron-deficient than males as observed in other studies [31–34], and these gender-specific differences persisted up to about 3 years of age. In agreement with previous studies [35–37], underweight children had increased ID and expanded erythropoiesis, suggesting that improving the nutritional status of children may also help to address ID and anemia.

Inflammation and malaria parasitemia substantially altered measures of iron status in agreement with previous studies [10, 38, 39]. We found that both inflammation and malaria were independently associated with increased levels of ferritin, thus, potentially leading to children being misclassified as iron replete. We therefore redefined ID by correcting ferritin levels for the effects of inflammation and malaria using regression correction, as previously proposed by BRINDA [10]. The strength of this approach is that it accounts for continuous measures of inflammation as opposed to the arbitrary cut-off points used by the WHO [7]. Compared to the inflammation and malaria-corrected definition of ID, the WHO definition substantially underestimated the prevalence of ID among children living in sub-Saharan Africa. This underestimate was particularly higher after adjusting for inflammation only and additional regression correction for malaria resulted in a more modest increase in the underestimate. The independent effect of inflammation on ferritin levels was also greater than that of malaria. Using a similar approach, Namaste et al. observed similar absolute percentage increases (of up to 27%) in children misclassified as iron replete using WHO-defined ID compared to regression-corrected ID [10].

The underestimate of prevalence of ID by the WHO was largest in cohorts that had a high burden of malaria and other infections, for example, 27.0% and 21.4% of Burkinabe and Kenyan children, respectively, were misclassified as iron replete compared to 8.5% of South African children. The unidentified burden of ID increased with age in line with increasing prevalence of malaria parasitemia, and we observed higher ferritin levels among children with malaria parasitemia at every decile of CRP. Malaria parasitemia also increased ferritin levels independently of inflammation in multivariable analyses. In agreement, a study in Burkinabe children found that adjustment for asymptomatic malaria, in addition to inflammation, led to a 11.9% absolute increase in the prevalence of ID [40]. Taken together, these findings indicate that both inflammation and malaria parasitemia should be accounted for in population estimates of prevalence of ID in African children.

We then evaluated the diagnostic utility of uncorrected iron biomarkers in predicting ID regression-corrected for inflammation and malaria. A TSAT < 11% best predicted regression-corrected ID indicating its potential usefulness in estimating the prevalence of ID in our study populations. TSAT is calculated from measured serum iron and either transferrin or UIBC, all of which have standard assays that are easy and inexpensive to perform. TSAT< 11% performed well over a range of CRP concentrations, in children with both malaria and inflammation, and across populations. In support of our findings, Aguilar et al. showed that TSAT had a high sensitivity (81%) in predicting bone marrow ID in 180 anemic (hemoglobin < 11 g/dL) children in Mozambique although specificity was low (40%) and an optimal cut-off was not derived [38]. In contrast, another study in Malawian children showed limited value of TSAT in diagnosing bone marrow iron stores in severely anemic children (hemoglobin < 5 g/dL) [25]. Other iron markers did not perform as well as TSAT in predicting corrected ID, for example sTfR concentrations had very low sensitivity (43%). The WHO recommends iron supplementation in populations where the prevalence of anemia is ≥ 40% [41]; however, we found that hemoglobin concentrations had very low specificity (42%) for predicting corrected ID probably because of the multifactorial etiology of anemia in African children [15]. Based on this guideline, all children in our study populations would have received iron, although approximately half were iron replete. Therefore, TSAT may be a better marker than hemoglobin for determining the prevalence of ID in African children although a more sensitive and specific marker is needed.

There were a number of important limitations of our study. The cross-sectional nature of our data limited us from analyzing longitudinal effects of inflammation, malaria, and nutritional status on iron status. Moreover, we did not measure α-1-acid glycoprotein (AGP), which has been shown to be a better marker for adjusting for inflammation in regression-correction analyses [10]. Nevertheless, unlike AGP, CRP is more widely measured and international reference standards are available. We used ID regression-corrected for inflammation and malaria as the gold standard although this method is yet to be validated, for example by either comparing prevalence estimates of regression modeling before and during/after infections or with bone marrow ID. We used a ferritin-based definition of ID since other iron biomarkers have less standardized assays and less well-established cut-offs for ID. Another limitation of our study was that TSAT, the best performing marker in predicting regression-corrected ID, was not available for Ugandan and South African children, and although it outperformed all other iron markers, it had an AUC of only 0.77.

## Conclusions

In this large-scale study including more than 4800 children in five countries across Africa, we explored a wide range of iron biomarkers to more accurately estimate prevalence of ID in countries with a high burden of childhood infections including malaria. There has been a long-standing concern regarding the challenge of using iron biomarkers to accurately estimate prevalence of ID in African populations [4]. In this study, we found that after accounting for the effects of inflammation and malaria on ferritin levels the prevalence of ID was substantially higher in African children than currently estimated by the WHO. Of the measured iron biomarkers, TSAT was the best predictor of ID determined by the gold standard of regression correction and may be useful in estimating prevalence of ID to guide planning and implementation of interventions, since the regression-correction approach would not be practical for programmatic screening of children in routine care. Further research is required for better interpretation of existing iron biomarkers and to identify newer ones that are not altered by malaria and other infections.

## Supplementary information

Additional file 1: **Table S1.** Laboratory assays for iron and inflammatory biomarkers by study site.

Additional file 2: **Table S2.** Associations between age, sex, inflammation, malaria, and nutritional status.

Additional file 3: **Table S3.** Regression-corrected and uncorrected prevalence of iron deficiency defined by additional iron biomarkers by study site.

Additional file 4: **Figure S1.** Multivariable regression models of the predictors of iron biomarkers with additional adjustment for underweight. Error bars indicate 95% confidence intervals and values indicate effect size (95% CI). Inflammation was defined as C-reactive protein > 5mg/L or α1-antichymotrypsin > 0.6g/dL (in The Gambia). Malaria was defined as *P. falciparum* parasitemia. BIS, body iron stores; sTfR: soluble transferrin receptor; TSAT, transferrin saturation; ZPP, zinc protoporphyrin (measured in The Gambia only). Underweight data were missing in South Africa.

Additional file 5: **Figure S2.** Comparison of prevalence of iron deficiency after additional regression-correction for age, sex and underweight. Graph letter ‘a’ indicates prevalence using WHO definition, ‘b’ correcting for malaria only, ‘c’ correcting for inflammation only, ‘d’ correcting for both malaria and inflammation, and ‘e’ correcting for malaria, inflammation, age, sex, and underweight. Error bars indicate 95% confidence intervals. No malaria in South Africa and anthropometry data were unavailable.

Additional file 6: **Figure S3.** Relationship between estimated prevalence of iron deficiency and inflammation / malaria. The graph shows the prevalence of iron deficiency in children with and without inflammation and / or malaria. Error bars indicate 95% confidence intervals. Inflammation was defined as C-reactive protein > 5mg/L or α1-antichymotrypsin > 0.6g/dL (in The Gambia). Low ferritin was defined as ferritin <12 μg/L in children <5 years or <15 μg/L in children ≥5 years. WHO adjustment involved using a higher cut-off (30 μg/L) of ferritin for children with inflammation according to WHO-defined ID. Malaria was defined as *P. falciparum* parasitemia. Regression-correction uses ferritin levels corrected for the effects of inflammation and malaria in defining ID. TSAT, transferrin saturation.

Additional file 7: **Figure S4.** Meta-analysis of optimal cut-offs of transferrin saturation in predicting iron deficiency regression-corrected for inflammation and malaria. Corrected iron deficiency was defined using the WHO definition with ferritin levels that were adjusted for malaria and inflammation using regression-correction. ES, effect size.

## Abbreviations

AGP: α-1-Acid glycoprotein
AUC: Area under curve
BIS: Body iron stores
BRINDA: Biomarkers Reflecting Inflammation and Nutritional Determinants of Anemia
CRP: C-reactive protein
EDTA: Ethylenediaminetetraacetic acid
EPI: Expanded Programme on Immunization
G6PD: Glucose-6-phosphate dehydrogenase
ID: Iron deficiency
IDA: Iron deficiency anemia
ROCs: Receiver operating characteristics curves
sTfR: Soluble transferrin receptor
TSAT: Transferrin saturation
UIBC: Unsaturated iron-binding capacity
WHO: World Health Organization
YLD: Years lived with disability
ZPP: Zinc protoporphyrin

## References

[1] Kassebaum NJ, Jasrasaria R, Naghavi M, Wulf SK, Johns N, Lozano R (2014). A systematic analysis of global anemia burden from 1990 to 2010. Blood J.

[2] GBD-2016-Disease-and-Injury-Incidence-and-Prevalence-Collaborators (2017). Global, regional, and national incidence, prevalence, and years lived with disability for 328 diseases and injuries for 195 countries, 1990–2016: A systematic analysis for the Global Burden of Disease Study 2016. Lancet.

[3] Mccann JC, Ames BN (2007). An overview of evidence for a causal relation between iron deficiency during development and deficits in cognitive or behavioral function. Am J Clin Nutr.

[4] Suchdev PS, Williams AM, Mei Z, Flores-ayala R, Pasricha S, Rogers LM (2017). Assessment of iron status in settings of inflammation : challenges and potential approaches. Am J Clin Nutr.

[5] Northrop-Clewes CA (2008). Interpreting indicators of iron status during an acute phase response--lessons from malaria and human immunodeficiency virus. Ann Clin Biochem.

[6] Phiri KS, Calis JCJ, Kachala D, Borgstein E, Waluza J, Bates I (2009). Improved method for assessing iron stores in the bone marrow. J Clin Pathol.

[7] World Health Organization. Serum ferritin concentrations for the assessment of iron status and iron deficiency in populations. Vitamin and Mineral Nutrition Information System. World Health Organization. 2011. https://www.who.int/vmnis/indicators/serum_ferritin.pdf.

[8] World Health Organization and Centers for Disease Control (CDC). Assessing the iron status of populations: including literature reviews: report of a Joint World Health Organization/Centers for Disease Control and Prevention Technical Consultation on the Assessment of Iron Status at the Population Level, Geneva, Switzer. 2007.

[9] Feelders RA, Vreugdenhil G, Eggermont AMM, Kuiper-Kramer PA, van Eijk HG, Swaak AJG (1998). Regulation of iron metabolism in the acute-phase response : interferon gamma and tumour necrosis factor alpha induce hypoferraemia, ferritin production and a decrease in circulating transferrin receptors in cancer patients. Eur J Clin Investig.

[10] Namaste SML, Rohner F, Huang J, Bhushan NL, Flores-ayala R, Kupka R (2017). Adjusting ferritin concentrations for inflammation : Biomarkers Reflecting Inflammation and Nutritional Determinants of Anemia ( BRINDA ) project. Am J Clin Nutr.

[11] Mast AE, Blinder MA, Gronowski AM, Chumley C, Scott MG (1998). Clinical utility of the soluble transferrin receptor and comparison with serum ferritin in several populations. Clin Chem.

[12] Menendez C, Quinto LL, Kahigwa E, Alvarez L, Fernandez R, Gimenez N (2001). Effect of malaria on soluble transferrin receptor levels in Tanzanian infants. Am J Trop Med Hyg.

[13] Verhoef H, West CE, Ndeto P, Burema J, Beguin Y, Kok FJ (2001). Serum transferrin receptor concentration indicates increased erythropoiesis in Kenyan children with asymptomatic malaria. Am J Clin Nutr.

[14] Rohner F, Namaste SM, Larson LM, Addo OY, Mei Z, Suchdev PS (2017). Adjusting soluble transferrin receptor concentrations for inflammation: Biomarkers Reflecting Inflammation and Nutritional Determinants of Anemia (BRINDA) project. Am J Clin Nutr.

[15] Foote EM, Sullivan KM, Ruth LJ, Oremo J, Sadumah I, Williams TN (2013). Determinants of Anemia among Preschool Children in Rural , Western Kenya. Am J Trop Med Hyg..

[16] Namaste SML, Aaron GJ, Varadhan R, Peerson JM, Suchdev PS. Methodologic approach for the Biomarkers Reflecting Inflammation and Nutritional Determinants of Anemia (BRINDA) project. Am J Clin Nutr. 2017;106(Suppl 1):333–47. 10.3945/ajcn.116.142273 PMC5490643 28615254

[17] Bejon P, Williams TN, Liljander A, Noor AM, Wambua J, Marsh K (2010). Stable and unstable malaria hotspots in longitudinal cohort studies in Kenya. PLoS Med.

[18] Elliott AM, Kizza M, Quigley MA, Ndibazza J, Nampijja M, Muhangi L (2007). The impact of helminths on the response to immunization and on the incidence of infection and disease in childhood in Uganda: design of a randomized, double-blind, placebo-controlled, factorial trial of deworming interventions delivered in pregnancy and early childhood. Clin Trials.

[19] Tiono AB, Nebie I, Anagnostou N, Coulibaly AS, Bowyer G, Lam E (2018). First field efficacy trial of the ChAd63 MVA ME- TRAP vectored malaria vaccine candidate in 5–17 months old infants and children. PLoS One.

[20] Nunes MC, Cutland CL, Jones S, Hugo A, Madimabe R, Simões EAF (2016). Duration of infant protection against influenza illness conferred by maternal immunization. JAMA Pediatr.

[21] Atkinson SH, Rockett K, Sirugo G, Bejon PA, Fulford A, O’Connell MA (2006). Seasonal childhood anaemia in West Africa is associated with the haptoglobin 2-2 genotype. PLoS Med.

[22] Wray K, Allen A, Evans E, Fisher C, Premawardhena A, Perera L (2017). Hepcidin detects iron deficiency in Sri Lankan adolescents with a high burden of hemoglobinopathy: A diagnostic test accuracy study. Am J Hematol.

[23] World Health Organization. Haemoglobin concentrations for the diagnosis of anaemia and assessment of severity. World Health Organization. 2011. https://www.who.int/vmnis/indicators/haemoglobin/en/.

[24] Cook JD, Flowers CH, Skikne BS (2003). The quantitative assessment of body iron. Blood..

[25] Phiri KS, Calis JCJ, Siyasiya A, Bates I, Brabin B, van Hensbroek MB (2009). New cut-off values for ferritin and soluble transferrin receptor for the assessment of iron deficiency in children in a high infection pressure area. J Clin Pathol.

[26] Yamanishi H, Iyama S, Yamaguchi Y, Kanakura Y, Iwatani Y (2003). Total iron-binding capacity calculated from serum transferrin concentration or serum iron concentration and unsaturated iron-binding capacity. Clin Chem.

[27] World Health Organization (2001). Iron deficiency anaemia: assessment, prevention, and control. A Guide for Programme Managers.

[28] World Health Organization. WHO Child Growth Standards: Methods and development. World Health Organization. 2006. https://www.who.int/childgrowth/standards/technical_report/en/.

[29] Youden WJ (1950). Index for rating diagnostic tests. Cancer..

[30] Snow RW, Sartorius B, Kyalo D, Maina J, Amratia P, Mundia CW (2017). The prevalence of Plasmodium falciparum in sub-Saharan Africa since 1900. Nature..

[31] Ziegler EE, Nelson SE, Jeter JM (2014). Iron stores of breastfed infants during the first year of life. Nutrients..

[32] Tamura T, Hou J, Goldenberg RL, Johnston KE, Cliver SP (1999). Gender difference in cord serum ferritin concentrations. Biol Neonate.

[33] Domellof M., Lonnerdal B., Dewey K. G., Cohen R. J., Rivera L. L., Hernell O. (2002). Sex Differences in Iron Status During Infancy. PEDIATRICS.

[34] Jaeggi T, Moretti D, Kvalsvig J, Holding PA, Tjalsma H, Kortman GAM (2013). Iron status and systemic inflammation, but not gut inflammation, strongly predict gender-specific concentrations of serum hepcidin in infants in rural Kenya. PLoS One.

[35] Pediatrics I (2003). Anemia and undernutrition among preschool children in Uttar Pradesh. India Indian Pediatr.

[36] Büyükkaragöz B, Akgun NA, Bulus AD, Aydogdu SD, Bal C (2017). Can soluble transferrin receptor be used in diagnosing iron deficiency anemia and assessing iron response in infants with moderate acute malnutrition?. Arch Argent Pediatr.

[37] Sumarmi S, Puspitasari N, Handajani R, Wirjatmadi B (2016). Underweight as a risk factor for iron depletion and iron-deficient erythropoiesis among young women in rural areas of East Java, Indonesia. Mal J Nutr.

[38] Aguilar R, Moraleda C, Quintó L, Renom M, Mussacate L, Macete E (2012). Challenges in the diagnosis of iron deficiency in children exposed to high prevalence of infections. PLoS One.

[39] Righetti Aurélie A, Wegmüller Rita, Glinz Dominik, Ouattara Mamadou, Adiossan Lukas G, N'Goran Eliézer K, Utzinger Jürg, Hurrell Richard F (2013). Effects of inflammation and Plasmodium falciparum infection on soluble transferrin receptor and plasma ferritin concentration in different age groups: a prospective longitudinal study in Côte d'Ivoire. The American Journal of Clinical Nutrition.

[40] Wessells KR, Hess SY (2014). Asymptomatic malaria infection affects the interpretation of biomarkers of iron and vitamin a status , even after adjusting for systemic inflammation , but does not affect plasma zinc concentrations among young children in Burkina Faso 1–3. J Nutr.

[41] World Health Organization. Guideline: Daily iron supplementation in infants and children. World Health Organization. 2016. https://www.who.int/nutrition/publications/micronutrients/guidelines/daily_iron_supp_childrens/en/. 27195348

