# Supplementary material for: Estimating the burden of iron deficiency among African children
Source: BMC Med. 2020 Feb 27;18:31. doi: 10.1186/s12916-020-1502-7 (PMC7045745; doi:10.1186/s12916-020-1502-7)
Supplement: Supplementary file 1 — Table S1. Laboratory assays for iron and inflammatory biomarkers by study site. [file 12916_2020_1502_MOESM1_ESM.docx]

**Table S1. Laboratory assays for iron and inflammatory biomarkers by study site.**

| **Site** | **Ferritin** | **Soluble transferrin receptor** | **Hepcidin** | **Serum iron** | **Transferrin** | **Unsaturated iron binding capacity** | **Zinc protoporphyrin** | **Hemoglobin/MCV** | **C-reactive protein** | **α_1_-antichymotrypsin** |
| --- | --- | --- | --- | --- | --- | --- | --- | --- | --- | --- |
| Kenya | Microparticle Enzyme Immunoassay (Abbott Architect, USA) | Human sTfR ELISA (BioVendor, Czech Republic) | DRG Hepcidin 25 [bioactive] high sensitive ELISA (DRG International, USA) | MULTIGENT iron calorimetric assay, Abbott Architect, USA | Chemiluminescent Microparticle Immunoassay (Abbott Architect, USA) | Not measured | Not measured | Coulter analyser (ACT5Diff, Beckman Coulter, USA) | MULTIGENT CRP Vario assay, (Abbott Architect, USA) | Not measured |
| Uganda | Microparticle Enzyme Immunoassay (Abbott Architect, USA) | Human sTfR ELISA (BioVendor, Czech Republic) | DRG Hepcidin 25 [bioactive] high sensitive ELISA (DRG International, USA) | Not measured | Chemiluminescent Microparticle Immunoassay (Abbott Architect, USA) | Not measured | Not measured | Coulter analyser (Beckman Coulter, Nyon, Switzerland) | MULTIGENT CRP Vario assay, (Abbott Architect, USA) | Not measured |
| Burkina Faso | Microparticle Enzyme Immunoassay (Abbott Architect, USA) | Human sTfR ELISA (BioVendor, Czech Republic) | DRG Hepcidin 25 [bioactive] high sensitive ELISA (DRG International, USA) | MULTIGENT iron calorimetric assay, Abbott Architect, USA | Chemiluminescent Microparticle Immunoassay (Abbott Architect, USA) | Not measured | Not measured | Coulter analyser (Beckman Coulter, USA) | MULTIGENT CRP Vario assay, (Abbott Architect, USA) | Not measured |
| South Africa | Microparticle Enzyme Immunoassay (Abbott Architect, USA) | Human sTfR ELISA (BioVendor, Czech Republic) | DRG Hepcidin 25 [bioactive] high sensitive ELISA (DRG International, USA) | Not measured | Chemiluminescent Microparticle Immunoassay (Abbott Architect, USA) | Not measured | Not measured | Not measured | MULTIGENT CRP Vario assay, (Abbott Architect, USA) | Not measured |
| The Gambia | Microparticle Enzyme Immunoassay (Abbott Architect, USA) | Quantikine sTfR ELISA kit, (R&D Systems, USA) | Hepcidin-25 [human] Enzyme Immunoassay Kit (Bachem, Switzerland) | Ferrozine-based photometry and colorimetry analyser (Hitachi 911, Hitachi, Tokyo, Japan) | Not measured | Ferrozine-based photometry and colorimetry analyser (Hitachi 911, Hitachi, Tokyo, Japan) | Aviv Biomedical Hematoflurometer (within 24 hours of collection) | Medonic CA 530 Oden 16 Hemoglobinometer | Not measured | Immunoturbidimetry, Cobas Mira Plus Bio-analyser, Roche |
