# Supplementary material for: Estimating the burden of iron deficiency among African children
Source: BMC Med. 2020 Feb 27;18:31. doi: 10.1186/s12916-020-1502-7 (PMC7045745; doi:10.1186/s12916-020-1502-7)
Supplement: Supplementary file 2 — Table S2. Associations between age, sex, inflammation, malaria, and nutritional status. [file 12916_2020_1502_MOESM2_ESM.docx]

**Table S2. Association between age, sex, inflammation, malaria, and nutritional status.**

|  | **Inflammation^*^** | | **Malaria^†^** | | **Underweight^‡^** | |
| --- | --- | --- | --- | --- | --- | --- |
|  | **OR (95% CI)** | **P value** | **OR (95% CI)** | **P value** | **OR (95% CI)** | **P value** |
| Age in years | 1.0 (0.9, 1.0) | 0.22 | 1.7 (1.6, 1.9) | <0.001 | 1.2 (1.04, 1.3) | 0.008 |
| Sex: Females | 1.0 (0.8, 1.1) | 0.61 | 0.9 (0.8, 1.1) | 0.41 | 1.0 (0.8, 1.2) | 0.64 |
| Underweight | 1.4 (1.1, 1.8) | 0.004 | 1.8 (1.4, 2.4) | <0.001 | N/A | N/A |
| Outcomes are columns labelled inflammation, malaria, stunting and underweight, while putative risk factors are rows including age, sex, and underweight. All models were adjusted for age, sex and cohort. N/A, not applicable  * Inflammation was defined as C-reactive protein > 5mg/L or α1-antichymotrypsin > 0.6g/dL (in The Gambia).  † Malaria was defined as *P. falciparum* parasitemia at any density.  **^‡^** Underweight was defined as weight for age z-score < -2. | | | | | | |
