# Supplementary material for: Estimating the burden of iron deficiency among African children
Source: BMC Med. 2020 Feb 27;18:31. doi: 10.1186/s12916-020-1502-7 (PMC7045745; doi:10.1186/s12916-020-1502-7)
Supplement: Supplementary file 3 — Table S3. Regression-corrected and uncorrected prevalence of iron deficiency defined by additional iron biomarkers by study site. [file 12916_2020_1502_MOESM3_ESM.docx]

**Table S3. Regression-corrected and uncorrected prevalence of iron deficiency defined by additional iron biomarkers by study site**.

|  | **Kenya** | | | **Uganda** | | | **Burkina Faso** | | | **South Africa** | | | **The Gambia** | | | **Pooled** | | |
| --- | --- | --- | --- | --- | --- | --- | --- | --- | --- | --- | --- | --- | --- | --- | --- | --- | --- | --- |
| **Definition** | **n** | **%** | **95% CI** | **n** | **%** | **95% CI** | **n** | **%** | **95% CI** | **n** | **%** | **95% CI** | **n** | **%** | **95% CI** | **n** | **%** | **95% CI** |
| ID - sTfR >8.3mg/L |  |  |  |  |  |  |  |  |  |  |  |  |  |  |  |  |  |  |
| Uncorrected | 1467 | 97.9 | 97.2, 98.6 | 1343 | 36.9 | 34.3, 39.5 | 342 | 94.2 | 91.7, 96.7 | 893 | 80.4 | 77.8, 83.0 | 661 | 1.7 | 0.7, 2.6 | 4706 | 63.4 | 62.0, 64.8 |
| Inflammation/malaria corrected | 1140 | 96.8 | 95.8, 97.9 | 1292 | 33.1 | 30.6, 35.7 | 302 | 94.4 | 91.8, 97.0 | 893 | 80.9 | 78.4, 83.5 | 659 | 1.5 | 0.6, 2.5 | 4286 | 59.5 | 58.0, 61.0 |
| Hepcidin<3.2µg/L |  |  |  |  |  |  |  |  |  |  |  |  |  |  |  |  |  |  |
| Uncorrected | 1373 | 31.7 | 29.2, 34.1 | 1333 | 26.0 | 23.6, 28.3 | 309 | 34.6 | 29.3, 40.0 | 878 | 22.9 | 20.1, 25.7 | 709 | 27.8 | 24.5, 31.1 | 4602 | 27.9 | 26.6, 29.2 |
| Inflammation/malaria corrected | 1075 | 51.3 | 48.3, 54.2 | 1282 | 35.2 | 32.6, 37.8 | 276 | 50.0 | 44.1, 55.9 | 878 | 31.4 | 28.4, 34.5 | 700 | 33.6 | 30.1, 37.1 | 4211 | 39.2 | 37.7, 40.7 |
| Depleted BIS (<0mg/kg)^*^ |  |  |  |  |  |  |  |  |  |  |  |  |  |  |  |  |  |  |
| Uncorrected | 1393 | 54.1 | 51.4, 56.7 | 1241 | 28.1 | 25.6, 30.6 | 322 | 55.9 | 50.4, 61.4 | 893 | 48.5 | 45.2, 51.8 | 660 | 7.3 | 5.3, 9.3 | 4509 | 39.1 | 37.7, 40.5 |
| Inflammation/malaria corrected | 1098 | 77.9 | 75.4, 80.3 | 1205 | 45.2 | 42.4, 48.0 | 291 | 82.5 | 78.1, 86.9 | 893 | 64.2 | 61.0, 67.3 | 658 | 12.8 | 10.2, 15.3 | 4145 | 55.4 | 53.9, 56.9 |
| Ferritin index>5.6^†^ |  |  |  |  |  |  |  |  |  |  |  |  |  |  |  |  |  |  |
| Uncorrected | 1392 | 96.4 | 95.4, 97.4 | 1241 | 48.9 | 46.1, 51.7 | 322 | 91.6 | 88.6, 94.7 | 893 | 85.1 | 82.8, 87.4 | 660 | 88.9 | 86.5, 91.3 | 4508 | 79.7 | 78.5, 80.8 |
| Inflammation/malaria corrected | 1098 | 96.5 | 95.4, 97.6 | 1205 | 57.7 | 54.9, 60.5 | 291 | 95.2 | 92.7, 97.7 | 893 | 90.3 | 88.3, 92.2 | 658 | 91.9 | 89.9, 94.0 | 4145 | 83.1 | 81.9, 84.2 |
| Iron deficiency anemia^‡^ |  |  |  |  |  |  |  |  |  |  |  |  |  |  |  |  |  |  |
| Uncorrected | 833 | 24.8 | 21.9, 27.8 | 1209 | 21.1 | 18.8, 23.4 | 309 | 31.1 | 25.9, 36.3 | n/a | n/a | n/a | 745 | 16.5 | 13.8, 19.2 | 3096 | 22.0 | 20.5, 23.5 |
| Inflammation/malaria corrected | 731 | 41.3 | 37.7, 44.9 | 1177 | 30.8 | 28.1, 33.4 | 290 | 55.5 | 49.8, 61.3 | n/a | n/a | n/a | 735 | 27.6 | 24.4, 30.9 | 2933 | 35.0 | 33.3, 36.8 |
| BIS, body iron stores; ZPP, zinc protoporphyrin; n/a, not available.  Regression-correction for inflammation and malaria were made on individual iron biomarkers and then used to define iron deficiency.  * Body iron stores were calculated using the ratio of soluble transferrin receptors (sTfR) and ferritin concentrations [25].  † Ferritin index defined as sTfR/log10 ferritin [26].  ‡ Iron deficiency anemia was defined as iron deficiency and anemia. | | | | | | | | | | | | | | | | | | |
