# Supplementary figures and images for: Estimating the burden of iron deficiency among African children
Source: BMC Med. 2020 Feb 27;18:31. doi: 10.1186/s12916-020-1502-7 (PMC7045745; doi:10.1186/s12916-020-1502-7)

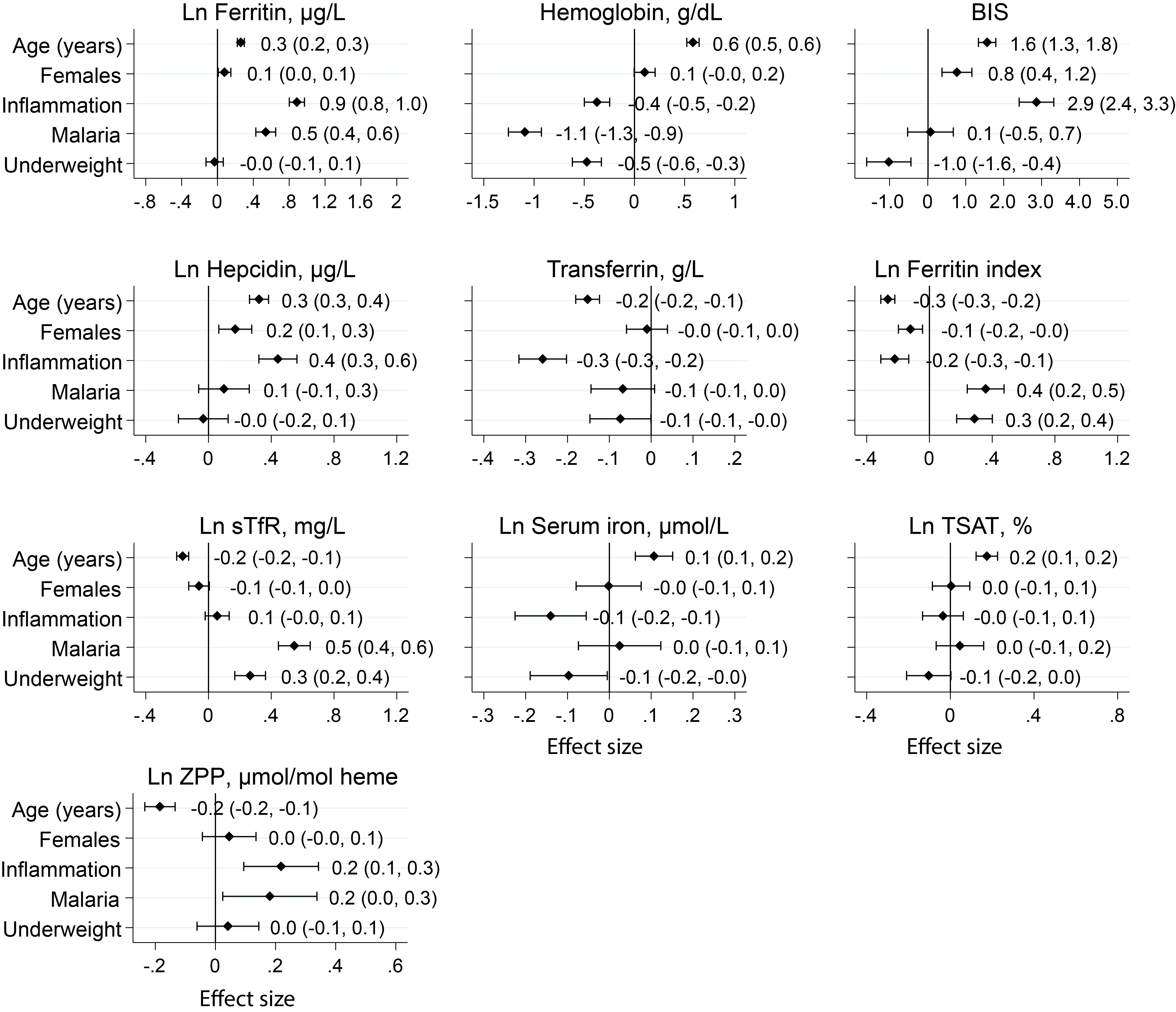

Supplement: Supplementary file 4 — Figure S1. Multivariable regression models of the predictors of iron biomarkers with additional adjustment for underweight. Error bars indicate 95% confidence intervals and values indicate effect size (95% CI). Inflammation was defined as C-reactive protein > 5mg/L or α1-antichymotrypsin > 0.6g/dL (in The Gambia). Malaria was defined as P. falciparum parasitemia. BIS, body iron stores; sTfR: soluble transferrin receptor; TSAT, transferrin saturation; ZPP, zinc protoporphyrin (measured in The Gambia only). Underweight data were missing in South Africa. [file 12916_2020_1502_MOESM4_ESM.png]

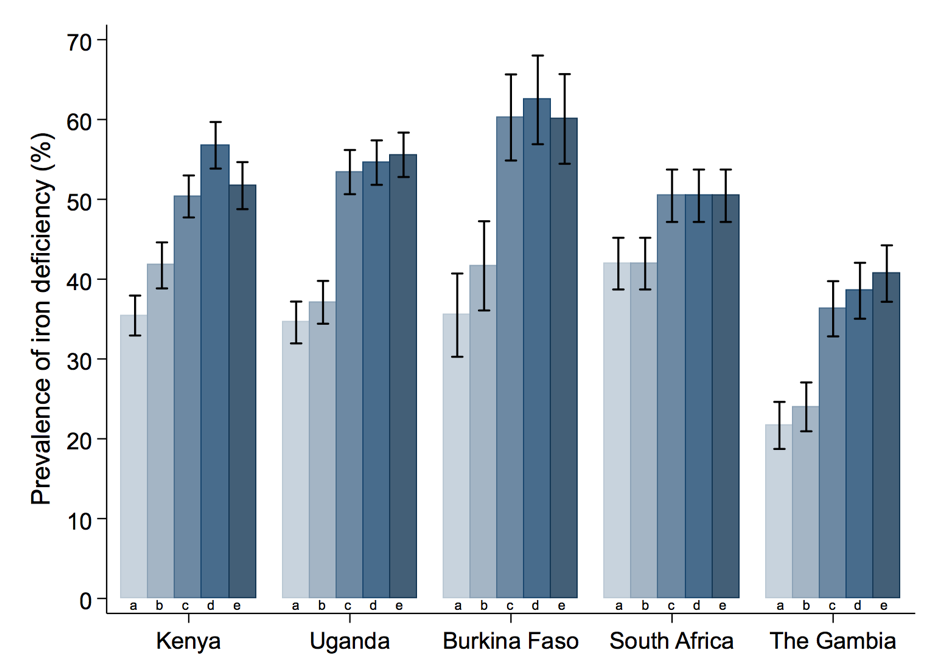

Supplement: Supplementary file 5 — Figure S2. Comparison of prevalence of iron deficiency after additional regression-correction for age, sex and underweight. Graph letter ‘a’ indicates prevalence using WHO definition, ‘b’ correcting for malaria only, ‘c’ correcting for inflammation only, ‘d’ correcting for both malaria and inflammation, and ‘e’ correcting for malaria, inflammation, age, sex, and underweight. Error bars indicate 95% confidence intervals. No malaria in South Africa and anthropometry data were unavailable. [file 12916_2020_1502_MOESM5_ESM.png]

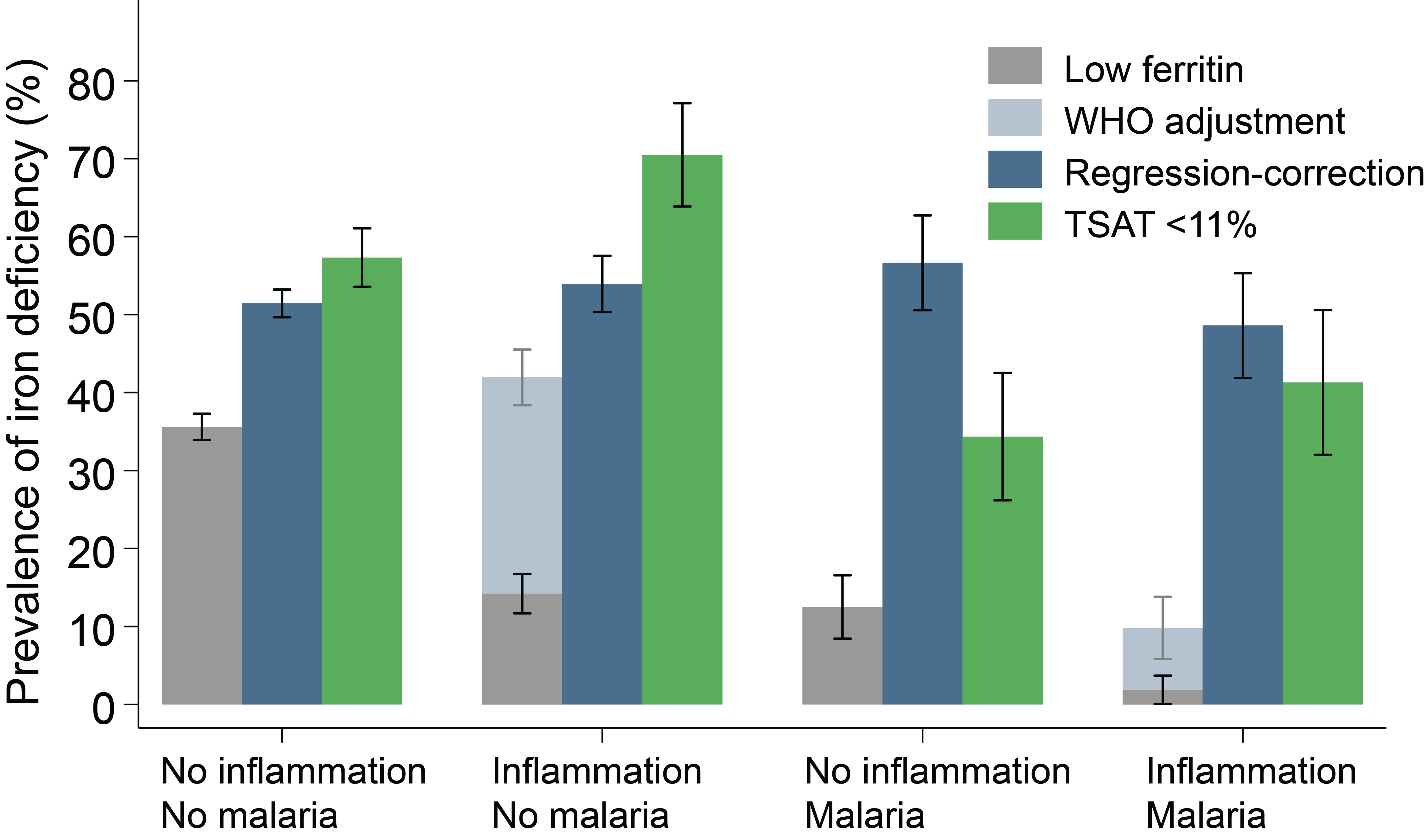

Supplement: Supplementary file 6 — Figure S3. Relationship between estimated prevalence of iron deficiency and inflammation / malaria. The graph shows the prevalence of iron deficiency in children with and without inflammation and / or malaria. Error bars indicate 95% confidence intervals. Inflammation was defined as C-reactive protein > 5mg/L or α1-antichymotrypsin > 0.6g/dL (in The Gambia). Low ferritin was defined as ferritin <12 μg/L in children <5 years or <15 μg/L in children ≥5 years. WHO adjustment involved using a higher cut-off (30 μg/L) of ferritin for children with inflammation according to WHO-defined ID. Malaria was defined as P. falciparum parasitemia. Regression-correction uses ferritin levels corrected for the effects of inflammation and malaria in defining ID. TSAT, transferrin saturation. [file 12916_2020_1502_MOESM6_ESM.png]

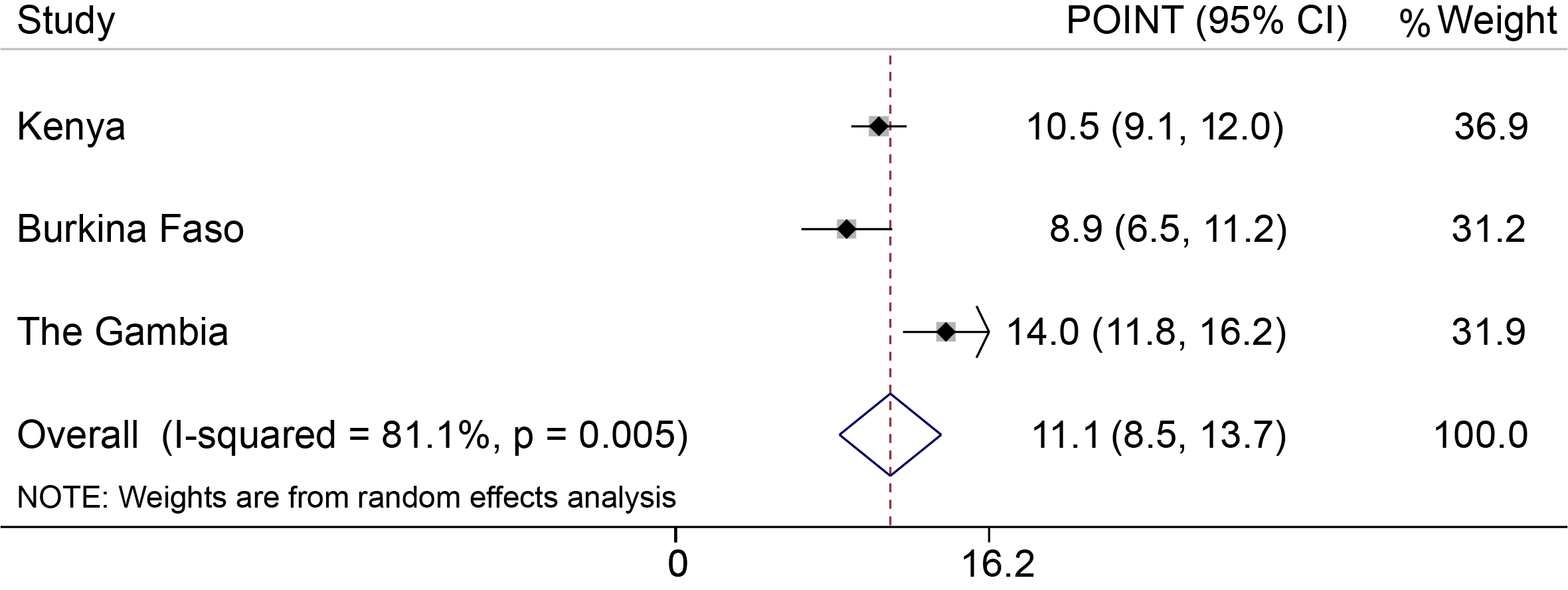

Supplement: Supplementary file 7 — Figure S4. Meta-analysis of optimal cut-offs of transferrin saturation in predicting iron deficiency regression-corrected for inflammation and malaria. Corrected iron deficiency was defined using the WHO definition with ferritin levels that were adjusted for malaria and inflammation using regression-correction. ES, effect size. [file 12916_2020_1502_MOESM7_ESM.png]
